# Effects of Maternal Homelessness, Supplemental Nutrition Programs, and Prenatal PM_2.5_ on Birthweight

**DOI:** 10.3390/ijerph16214154

**Published:** 2019-10-28

**Authors:** Jongeun Rhee, M. Patricia Fabian, Stephanie Ettinger de Cuba, Sharon Coleman, Megan Sandel, Kevin James Lane, Maayan Yitshak Sade, Jaime E. Hart, Joel Schwartz, Itai Kloog, Francine Laden, Jonathan I. Levy, Antonella Zanobetti

**Affiliations:** 1 Department of Environmental Health, T.H. Chan School of Public Health, Harvard University, Boston, MA 02115, USA; myitshak@hsph.harvard.edu (M.Y.S.); rejch@channing.harvard.edu (J.E.H.); joel@hsph.harvard.edu (J.S.); fladen@hsph.harvard.edu (F.L.); azanobet@hsph.harvard.edu (A.Z.); 2 Department of Environmental Health, School of Public Health, Boston University, Boston, MA 02115, USA; PFABIAN@bu.edu (M.P.F.); klane@bu.edu (K.J.L.); jonlevy@bu.edu (J.I.L.); 3 Department of Pediatrics, School of Medicine, Boston University, Boston, MA 02118, USA; sedc@bu.edu (S.E.d.C.); megan.sandel@bmc.org (M.S.); 4 Biostatistics and Epidemiology Data Analytics Center, School of Public Health, Boston University, Boston, MA 02118, USA; sharcole@bu.edu; 5 Department of Pediatrics, Boston Medical Center, Boston, MA 02118, USA; 6 Department of Medicine, Channing Division of Network Medicine, Brigham and Women’s Hospital and Harvard Medical School, Boston, MA 02115, USA; 7 Department of Epidemiology, T.H. Chan School of Public Health, Harvard University, Boston, MA 02115, USA; 8 Department of Geography and Environmental Development, Ben-Gurion University of the Negev, Beer Sheva, Israel; ikloog@bgu.ac.il

**Keywords:** maternal socioeconomic status, maternal homelessness, supplemental nutrition programs, PM_2.5_, birthweight

## Abstract

Few studies examined the impact of maternal socioeconomic status and of its combined effects with environmental exposures on birthweight. Our goal was to examine the impact of maternal homelessness (mothers ever homeless or who lived in shelters during pregnancy) and participation in the Special Supplemental Nutrition Program for Women, Infants and Children (WIC) during pregnancy in conjunction with air pollution exposure on birthweight in the Boston-based Children’s HealthWatch cohort from 2007 through 2015 (n = 3366). Birthweight was obtained from electronic health records. Information on maternal homelessness and WIC participation during pregnancy were provided via a questionnaire. Prenatal fine particulate matter (PM_2.5_) exposures, estimated at the subject’s residential address, were calculated for each trimester. We fit linear regression models adjusting for maternal and child characteristics, seasonality, and block-group-level median household income and examined the interactions between PM_2.5_ and each covariate. Prenatal maternal homelessness was associated with reduced birthweight (−55.7 g, 95% CI: −97.8 g, −13.7 g), while participating in WIC was marginally associated with increased birthweight (36.1 g, 95% CI: −7.3 g, 79.4 g). Only average PM_2.5_ during the second trimester was marginally associated with reduced birthweight (−8.5 g, 95% CI: −19.3, 2.3) for a 1 µg/m^3^ increase in PM_2.5_. The association of PM_2.5_ during the second trimester with reduced birthweight was stronger among non-Hispanic Black mothers and trended toward significance among immigrants and single mothers. Our study emphasizes the independent and synergistic effects of social and environmental stressors on birthweight, particularly the potentially protective effect of participating in WIC for vulnerable populations.

## 1. Introduction

Low birthweight is a leading cause of neonatal morbidity and mortality [1,2,3]. Maternal and environmental factors such as socioeconomic status, psychosocial stress, housing instability, and ambient air pollution have been independently associated with low birthweight [4,5,6,7,8,9,10,11,12,13,14,15]. However, few studies examined the impact of social stressors, e.g., individual and areal poverty, and their combined effects with environmental exposures on birthweight [16,17]. Investigating combined effects is important to identify the most vulnerable populations and optimize interventions to improve neonatal and child health. 

Housing instability is a social condition that may act as a severe stressor during pregnancy and affect infants’ birthweight [18]. Homeless women are more likely to be undernourished, experience abusive situations, have poor mental health, and be exposed to substance use during pregnancy [13,19,20]. Homeless pregnant women are also more likely to have lower educational attainment and to be younger, unmarried, Black, and participate in public assistance programs [13,14]. Maternal homelessness during pregnancy [14] or at the time of delivery [13] is associated with lower birthweight compared to the birthweight of infants of non-homeless women. Previously, our group also found that homelessness during pregnancy was associated with low birthweight, using data from the Children’s HealthWatch (CHW) cohort, collected in Baltimore, Boston, Little Rock, Minneapolis, and Philadelphia [15]. 

Several studies have shown that participation in the Special Supplemental Nutrition Program for Women, Infants and Children (WIC) is associated with an increase in birthweight of the infants of low-income pregnant women [21,22]. WIC is the United States Department of Agriculture’s (USDA) third largest nutrition assistance program and provides supplemental food to low-income pregnant and nursing women, infants, and children up to the age five. WIC serves approximately 7 million women and children per month [23]. In studies conducted in North and South Carolina, low-income mothers who participated in prenatal WIC services had lower rates of low birthweight [21] and delivered children with higher birthweights [22] compared to mothers who did not participate during pregnancy. 

Although many studies have shown associations between air pollution exposure and reduced birthweight [8,10,11,12,24], there is limited evidence of the interactions between air pollution exposure and socioeconomic status (SES). A study conducted in North Carolina found that an increase in PM_2.5_ during the gestational period reduced birthweight but had no interaction with SES [16]. Another study showed increased birthweight at greater distance from major highways. The protective effect was greater among residents with higher education [25]. Several studies investigating preterm birth found stronger associations with air pollution in low-SES neighborhoods [26,27,28]. However, no studies considered specific stressors or protective factors, including joint associations or interactions with housing instability or WIC participation as a marker of SES. 

In our study, we aimed to examine the independent effects of prenatal maternal homelessness, WIC participation during pregnancy, and fine particulate matter (PM_2.5_) on birthweight in a cohort of low-income urban children in Boston. We additionally examined the interactions between maternal and child characteristics, prenatal maternal homelessness, or WIC participation and prenatal PM_2.5_ exposure on birthweight. 

## 2. Materials and Methods

### 2.1. Study Population

The study population included children recruited to the Boston-based Children’s HealthWatch cohort from 2007 through 2015 (n = 3366). Children’s HealthWatch (http://www.childrenshealthwatch.org) is an ongoing collaborative research study monitoring the health and well-being of young children and their families in five cities across the United States [29] and includes clinical and interview data from urban emergency departments (EDs) and ambulatory care clinics [30]. In Boston, the survey was administered to caregivers of children seeking medical care for their children during an ED visit. Study research assistants obtained consent and determined eligibility. Eligibility criteria included the following: child age ≤48 months; residency in Massachusetts; caregiver ability to speak English or Spanish; respondent living in the child’s household [31]. Trained interviewers asked caregivers questions including their demographics, socioeconomic characteristics, smoking history, and child health and development [31]. From the Children’s HealthWatch survey, we extracted year of enrollment, maternal age, maternal height and weight (to calculate maternal body mass index (BMI)), maternal race/ethnicity (Hispanic, non-Hispanic Black, non-Hispanic white, and other), maternal nativity (US born, immigrant), education (some high school or less, high school, technical school/college graduate/master’s level or higher), smoking history (smoked cigarettes or used any other tobacco products in the last 5 years), marital status (single, married/partnered/cohabitating, separated/divorced/widowed), maternal prenatal homelessness during pregnancy (ever homeless or lived in shelter during pregnancy with this child), child health insurance status (no insurance, public, private), and participation in WIC during pregnancy. Approval was obtained from the Boston University Medical Campus Institutional Review Board (H-34069).

### 2.2. Birthweight Outcome Data

Survey data were matched to the electronic health record (EHR) based on medical record number, with date of the Children’s HealthWatch (CHW) interview, sex, and date of birth as confirmation factors. From the her, we extracted the following information: birthweight (g), residential address, gestational age (weeks), child sex, and child date of birth. Children who missed EHR birthweights (42%) and gestational ages (44%), were assigned birthweight and gestational age information from the CHW survey data. These two sources of data had high correlation coefficients—birthweight (0.97) and gestational age (0.95). When CHW survey and EHR values were different, we used the EHR value. 

### 2.3. Air Pollution and Covariate Data

Maternal residential addresses obtained from the EHR were cleaned and geocoded to parcels using ArcMAP v.10.6 (ESRI, ArcGIS, Redlands, CA, USA). Less than 1% of all addresses listed in the EHR were either missing or listed as a P.O. Box. Nearly 3.6% were unmatched among the remaining addresses that were included in the geocoding process. We assigned daily PM_2.5_ concentration to each residential address at birth using a spatio-temporally resolved hybrid model with a 1 × 1 km resolution [32], which has been validated and used in previous studies [9,33,34]. Briefly, daily PM_2.5_ was predicted using a mixed-effects model with satellite-retrieved aerosol optical depth (AOD) measurements, monitoring data, land use data, and meteorological variables [32]. The model performance was excellent (mean out of sample R^2^ = 0.87), with very little bias in the predicted concentration (slope of predictions versus withheld observations = 0.99). We assigned daily PM_2.5_ concentration of the closest grid cell to each geocoded residential address. Then, we calculated the average PM_2.5_ concentration during the 1st, 2nd, and 3rd trimesters using date of birth and gestational age (in weeks). Additionally, we assigned block-group-level median household income to each geocoded address using American Community Survey data from 2007 to 2015. 

### 2.4. Analytical Sample Selection

**Figure 1:**
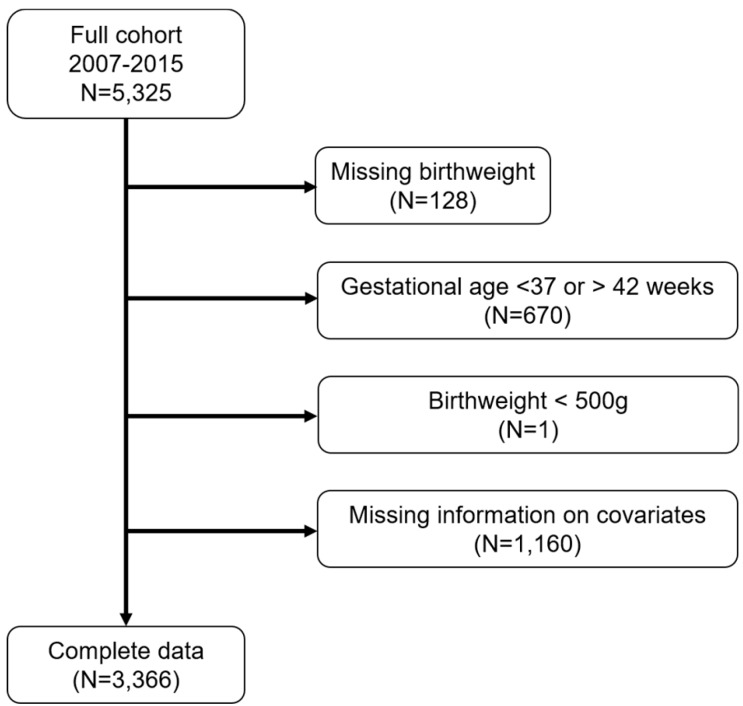
Number of Boston-based Children’s HealthWatch study subjects excluded from the final analytical sample.

We excluded subjects with missing birthweight information (n = 128), births with gestational age <37 or >42 weeks (n = 670), and children with birthweight <500 g (n = 1). We also excluded subjects with no information on the following covariates: maternal age, BMI, race/ethnicity, nativity, education, smoking history, child insurance status, marital status, and child sex (Figure 1).

### 2.5. Statistical Analysis

We examined the summary characteristics for prenatal maternal homelessness, WIC, prenatal PM_2.5_, birthweight, and other covariates. Spearman correlation tests were performed between variables to assess for potential collinearity. We fit a linear regression model including average PM_2.5_ values during the 1st, 2nd, 3rd trimesters, maternal homelessness, and WIC support, adjusting for maternal age, BMI, race/ethnicity, nativity, education, smoking, insurance, marital status, child gestational age and sex, seasonality, and block-group-level median household income. We also fit the same model but included natural cubic splines for average PM_2.5_ values during the 1st, 2nd, 3rd trimesters (3 degrees of freedom) instead of linear terms to examine the concentration–response relationship. Since 30% of the information on individual household incomes was missing, we did not include this in the analysis. Seasonality was attributed from the month of date of birth. We created a categorical variable with indicators for four seasons. We included all trimesters’ PM_2.5_ exposures in the same model to evaluate windows of vulnerability on birthweight. To examine effect modifications, we included interactions between PM_2.5_ and each covariate, including maternal and child characteristics, prenatal maternal homelessness, or WIC participation. Then, we computed the effect of PM_2.5_ for each category of the modifier. All analyses were performed in RStudio Version 1.2.1335 (RStudio Team (2018). RStudio: Integrated Development for R. RStudio, Inc., Boston, MA URL http://www.rstudio.com/). All testing was done with a two-sided alpha level of 0.05.

## 3. Results

We included 3366 participants in our analysis (Figure 1). The median birthweight was 3272 g (Table 1). The majority of mothers in the cohort were non-Hispanic Black (50%), followed by Hispanic (33%). Nearly 40% of mothers were immigrants, and 84% participated in WIC during pregnancy. Most mothers did not smoke in the past 5 years (74%), and nearly half of the mothers attained more than a high school education (47%). Half of the study subjects reported that their household incomes were below $20,000 (data not shown). The majority of children had public health insurance (86%). The median PM_2.5_ concentrations across each trimester were similar (1st: 9.1 µg/m^3^, 2nd: 9.2 µg/m^3^, 3rd: 8.9 µg/m^3^).

**Figure 2:**
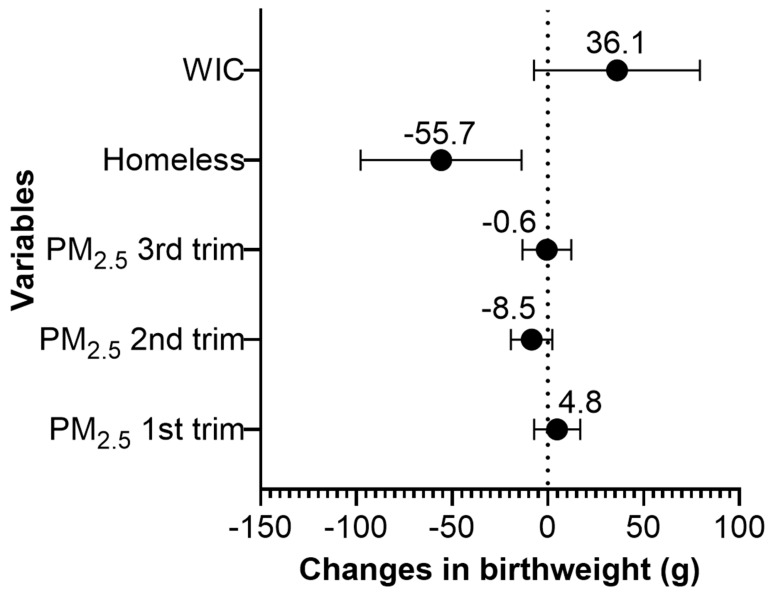
Change in birthweight (g) for a 1 µg/m^3^ increase in the average PM_2.5_ during the 1st, 2nd, and 3rd pregnancy trimesters, for prenatal homelessness, and for the participation status in the Special Supplemental Nutrition Program for Women, Infants and Children (WIC) in the Boston-based Children’s HealthWatch cohort (N = 3366). Note: Adjusted for maternal age, body mass index (BMI), race/ethnicity, nativity, education, smoking history, insurance, marital status, child gestational age and sex, seasonality, and median household income.

We found that maternal homelessness during pregnancy was associated with 56 g lower birthweight (95% CI: −97.8 g, −13.7 g) (Figure 2, Table S1). Pregnant women who participated in WIC during pregnancy delivered children with 36 g higher birthweight compared to women who did not participate in the program (95% CI: −7.3 g, 79.4 g); however, the association was only marginally significant. A 1 µg/m^3^ increase in average PM_2.5_ during the 2nd trimester was marginally associated with 9 g lower birthweight (95% CI: −19.3, 2.3). The average PM_2.5_ levels during the 1st or 3rd trimester were not associated with birthweight. The concentration–response plots for average PM_2.5_ during the 1st, 2nd, and 3rd trimesters showed linear or almost linear relationships with birthweight (Figures S1–S3).

**Figure 3:**
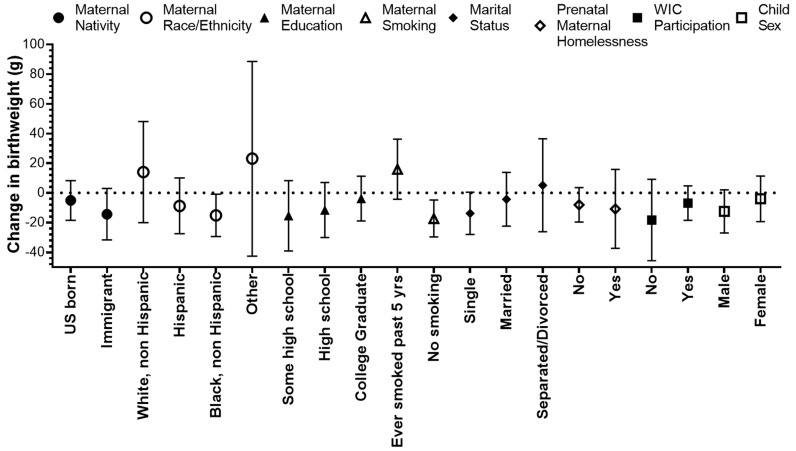
Change in birthweight (g) for a 1 µg/m^3^ increase in the average PM_2.5_ during the 2nd trimester, stratified by maternal and child characteristics in the Boston-based Children’s HealthWatch cohort.

We found that the effect of PM_2.5_ during the 2nd trimester on reduced birthweight was stronger among non-Hispanic Black mothers and was marginally significant among immigrants and single mothers (Figure 3, Table S2). The average birthweight of male children was higher than that of female children (3345 g vs. 3227 g); however, the effect of PM_2.5_ on reduced birthweight was stronger for males (Figure 3, Table S2). PM_2.5_ exposure was associated with increased birthweight among mothers who smoked in the past 5 years compared to mothers who did not smoke. We did not find significant interactions between PM_2.5_ exposure during the 2nd trimester and maternal homelessness or WIC participation. 

## 4. Discussion

In this study, we found WIC participation during pregnancy can potentially benefit fetal growth as exhibited by higher birthweight, while being homeless or having increased exposure to air pollution during the 2nd trimester decreased birthweight, especially among immigrants, non-Hispanic Black mothers, and single mothers.

We focused on an under-investigated population at high risk of poor outcomes in an urban setting, including 40% immigrant mothers and 50% non-Hispanic Black mothers, and found that the impact of prenatal PM_2.5_ on reduced infant birthweight was stronger among these groups. Previous findings did not explicitly examine maternal immigration status with detailed ethnicity and race data, which are important for public health policy to identify target populations for assistance and intervention [8,10,16]. Others have shown that rates of low birthweight are lower among infants of Mexican-born Latinas than among infants of their US-born counterparts (“*birthweight paradox*”), despite the fact that recently arrived Latina immigrants are less likely to have received adequate prenatal care [35,36,37]. In our cohort, we similarly found higher birthweight on average among immigrant mothers (3353 g) compared to US born mothers (3251 g). Also, immigrant Hispanic mothers (3364 g) delivered higher birthweight infants on average than US born Hispanic mothers (3237 g). However, the effect of PM_2.5_ on reduced birthweight was worse among immigrant mothers. Bell et al. (38) found the effect of PM_2.5_ on reduced birthweight was stronger among infants of Black mothers (−22.6 g per IQR, 95% CI: −29.3, −15.9) compared to those of white mothers (−14.7 g, 95% CI: −17.3, −12.0) (ethnicity not specified). Morello-Frosch et al. [38] also found that PM_2.5_ effect estimates for decreases in average birthweight were strongest for African Americans. Although Gray et al. [16] did not find interactions between SES and air pollution, they found that non-Hispanic Black, Hispanic mothers with lower educational attainment, and mothers living in low-SES neighborhoods had infants with reduced birthweights compared to non-Hispanic white, highly educated mothers, and those living in moderate-income neighborhoods. A Chinese study [39] showed a stronger effect of air pollutants on preterm birth among rural-to-urban migrants, who had lower incomes and educational attainment compared to local residents. Disturbingly, we found PM_2.5_ exposure was associated with increased birthweight for mothers who smoked in the past 5 years and with reduced birthweight for non-smoker mothers. However, these findings should be interpreted with caution. We had information on mother’s smoking status during pregnancy; however, due to extensive missing data (80%), we used mother’s five-year smoking history instead, which may or may not have been concurrent with pregnancy. Our findings may be related to using crude smoking information and significant exposure misclassification because of self-reporting. 

Maternal homelessness during pregnancy is associated with reduced birthweight [13,14], however, WIC participation is associated with increased birthweight among low-income [21,22] and homeless [40] pregnant women. We found that mothers who were homeless or lived in a shelter during pregnancy had infants with a birthweight 56 g lower on average than that of infants of mothers who were not homeless. Similarly, our group found that homelessness during pregnancy was associated with a 53 g lower adjusted mean birthweight (*p* = 0.08), using data from the CHW cohort, collected in Baltimore, Boston, Little Rock, Minneapolis, and Philadelphia [15]. Richards et al. [14] found that maternal homelessness was associated with a 24 g decrease in infant birthweight compared to the birthweight of infants of non-homeless women. They used the Pregnancy Risk Assessment Monitoring System (PRAMS) data from 31 states/cities, which included around 4% homeless women. Compared to Richards et al. [14], nearly 16% of mothers in our cohort reported that they were homeless during pregnancy. Another study investigating low birthweight (under 2000 g) [13] found a 6.9 times greater odds of low birthweight (95% CI: 2.4, 20.0) for mothers who were homeless at the time of delivery. From PRAMS data, Richards [40] et al. compared homeless women who participated in WIC during pregnancy and homeless women who did not participate in the program and found that the mean birthweight for homeless pregnant women in the WIC program was significantly higher (3182 g vs. 3109 g). In our study, we found WIC participation was marginally associated with a 36 g increased birthweight for multi-ethnic low-income mothers. Sonchak et al. [22] found in an unadjusted model that WIC participation was associated with a 51.6 g increase in birthweight for mothers with at least two births financed by Medicaid. After accounting for gestation, WIC participation was associated with a 7.6 g increase in birthweight [22]. They also found stronger effects of WIC on birthweight among Black mothers (63.0 g) compared to white mothers (38.6 g), even though the average birthweight was lower for Black mothers (3036.3 g, white mothers: 3251.0 g) [22]. In our cohort, the average birthweights for non-Hispanic Black mothers were lower (3268 g) than those for non-Hispanic white mothers (3412 g) and were similar to those for Hispanic mothers (3298 g). Since Sonchak et al. [22] did not sub-stratify race with ethnicity, we acknowledge that the populations are not entirely comparable. 

The relationship between birthweight and exposure to PM_2.5_ by exposure window is inconsistent [8,10,24,38]. Bell et al. [10] found that the 2nd and 3rd trimesters were the most important trimesters of exposure related to reduced birthweight in Massachusetts and Connecticut, whereas Morello-Frosch et al. (8) found that among California children, exposure in the 1st trimester had the greatest effect. Another study in California [38] found the effect of PM_2.5_ during the 2nd trimester on birthweight was the greatest (−46.6 g for PM_2.5_ > 18.4 µg/m^3^ vs. PM_2.5_ < 11.9 µg/m^3^) compared to the effect of other trimesters. A European cohort study (ESCAPE) found similar ORs for low birthweight across exposure windows [24]. In our study, we found exposure to PM_2.5_ during the 2nd trimester was marginally associated with reduction in birthweight. Different results across studies could be due to different exposure measurements and different correlations of the exposure between trimesters. Harris et al. [41] showed that the trimester associated with low birthweight varied according to differences in the spatial scale of PM_2.5_ exposures (grid-level vs. county-level), possibly because of exposure misclassification. Similarly, the size of effect estimates can be affected by using different exposure metrics [42]. We used predicted 1 × 1 km-resolution PM_2.5_ concentrations in our analysis; however, previous studies used county-level data [10], distance from the monitor [8], nearest monitor [38], and land-use regression models [24]. Correlations between trimester-specific exposures seem different by data source. In our study, the average PM_2.5_ during the 1st and 3rd trimesters had a relatively high correlation (Spearman correlation coefficient: 0.5); however, other combinations had low correlations. The California study found high correlation coefficients between the 1st and the 2nd trimester (0.6) and between the 2nd and the 3rd trimesters (0.6) [38]. 

Biologic mechanisms whereby PM affects birth outcomes are not well understood; however, multiple plausible hypotheses have been proposed: oxidative stress [43], pulmonary and placental inflammation, blood coagulation, endothelial dysfunction, and blood pressure elevation [44]. Prenatal exposure to air pollutants can affect fetal growth directly by passing across the placenta or indirectly by worsening maternal health during pregnancy [45]. PM is a complex mixture of chemical compounds, and each constituent may affect fetal growth through different biological pathways. Transplacental exposure to combustion-derived particles or PM metal-related components, including aluminum and titanium, induces oxidative stress [43], which may adversely affect the early development of the human embryo [46]. Oxidative stress-induced DNA damage may disrupt DNA transcription, which could increase the number of placental DNA adducts [44]. PM also absorbs and transports polycyclic aromatic hydrocarbons (PAHs), which may increase DNA adducts, resulting in reduced birthweight [47,48]. Inhalation of PM during pregnancy can induce acute placental and pulmonary inflammation [12,44]. Inflammation could reduce the perfusion of the placenta, which may result in impaired transplacental nutrient exchange [44]. 

We recognize a few limitations in our study. First, we cannot rule out recall bias as we used self-reported homelessness and WIC participation during pregnancy. However, systematic bias appears unlikely, and the effect estimate may be larger without exposure misclassification. Second, in our study, we did not have information on how the timing (or duration) of being homeless in combination with participating in WIC led to worse or better birth outcomes. Third, we adjusted for block-group median household income instead of individual household income in the analysis, due to 30% of missing information. Given that half of study subjects had low incomes (household incomes below $20,000), we assumed that variation in individual income was not substantial. Also, neighborhood SES is an important measure to capture birth outcome disparities [49].

Our study has numerous strengths. We investigated an understudied population at high risk for poor outcomes, including racial/ethnic-minority, immigrant, and low-income mothers and their children. In our cohort, half of the mothers were non-Hispanic Black, 40% of them were immigrants, and nearly all had public health insurance. Many prior studies of risk factors for low birthweight focused on lower risk non-Hispanic white populations. We demonstrated the importance of WIC for healthy birthweights and provided critical evidence for public health policy regarding which sub-populations need to be targeted to improve fetal growth. By investigating a multi-ethnic and highly vulnerable population of mothers, our research findings could directly inform health care decisions at the individual and population levels. In addition, this study is the first to examine the effects of simultaneous interactions between prenatal maternal homelessness, WIC participation, and air pollution on birthweight, reflecting a more real-life combination of exposures. By utilizing predicted air pollution data, we were able to include subjects who did not live close to monitoring sites to examine the effects of air pollution on birthweight. Finally, trimester-specific air pollution estimations provided us insight into a critical prenatal window associated with reduced birthweight. 

## 5. Conclusions

Our study emphasizes the independent and combined effects of maternal social and environmental stressors on birthweight, including homelessness and exposure to PM_2.5_, as well as the potential protective effect of participating in WIC during pregnancy. Importantly, even within a predominantly low-income population, the association of prenatal PM_2.5_ with birthweight was stronger among non-Hispanic Black mothers and marginally significant among immigrants and single mothers, which indicates the need for special attention to the circumstances of these groups of mothers. These findings can be used to better target health interventions and support policy change for pregnant mothers at high risk to maximize a healthy start for newborns.

## Figures and Tables

**Table 1:** Maternal and child characteristics of the Boston-based Children’s HealthWatch cohort (N = 3366).

Maternal Characteristics	Median (IQR) or N (%)
**Age (yr)**	27.0 (10.0)
**Race/ethnicity**	
Hispanic	1120 (33.3%)
Black, non Hispanic	1693 (50.3%)
White, non Hispanic	346 (10.3%)
Other	207 (6.1%)
**Nativity**	
US born	2072 (61.6%)
Immigrant	1294 (38.4%)
**Marital status**	
Single	1828 (54.3%)
Married/Partnered/Cohabitating	1158 (34.4%)
Separated/Divorced/Widowed	380 (11.3%)
**Education**	
Some high school or less	687 (20.4%)
High school	1084 (32.2%)
Tech School/College Graduate/Master’s	1595 (47.4%)
**Ever smoked in last 5 years**	
Yes	886 (26.3%)
No	2480 (73.7%)
**BMI (kg/m^2^)**	27.1 (8.3)
**Block group-level median household income ($)**	43,333 (30,253)
Average PM_2.5_ during entire pregnancy period (µg/m^3^)	9.3 (1.2)
Average PM_2.5_ during 1st trimester (µg/m^3^)	9.1 (2.2)
Average PM_2.5_ during 2nd trimester (µg/m^3^)	9.2 (2.1)
Average PM_2.5_ during 3rd trimester (µg/m^3^)	8.9 (2.1)
**Ever homeless or live in shelter during pregnancy with this child**	
Yes	524 (15.6%)
No	2842 (84.4%)
**Prenatal WIC participation with this child**	
Yes	2836 (84.3%)
No	530 (15.7%)
**Child characteristics**	**N (%)**
Birthweight (g)	3272 (630)
Gestational age (weeks)	39.6 (1.6)
**Sex**	
Male	1799 (53.4%)
Female	1567 (46.6%)
**Health insurance**	
Public	2903 (86.2%)
No insurance	103 (3.1%)
Private	360 (10.7%)

IQR: interquartile range, PM_2.5_: fine particulate matter.

## Supplementary Materials

The following are available online at https://www.mdpi.com/1660-4601/16/21/4154/s1, **Figure S1**: Concentration-response function of the exposure to average PM_2.5_ during 2nd trimester (µg/m^3^) on change in birthweight (g) in Boston-based Children’s HealthWatch cohort (N = 3366), **Figure S2**: Concentration-response function of the exposure to average PM_2.5_ during 1st trimester (µg/m^3^) on change in birthweight (g) in Boston-based Children’s HealthWatch cohort (N = 3366), **Figure S3**: Concentration-response function of the exposure to average PM_2.5_ during 3rd trimester (µg/m^3^) on change in birthweight (g) in Boston-based Children’s HealthWatch cohort (N = 3366), **Table S1**: Change in birthweight (g) for a 1 µg/m^3^ increase in the average PM_2.5_ during the 1st, 2nd, and 3rd pregnancy trimesters and for prenatal homelessness and the Special Supplemental Nutrition Program for Women, Infants and Children (WIC) participation status in Boston-based Children’s HealthWatch cohort (N = 3619). **Table S2**: Change in birthweight (g) for a 1 µg/m^3^ increase in the average PM_2.5_ during the 2nd trimester across maternal and child characteristics in Boston-based Children’s HealthWatch cohort (N = 3619).
