# Supplementary material for: Effects of Maternal Homelessness, Supplemental Nutrition Programs, and Prenatal PM2.5 on Birthweight
Source: Int J Environ Res Public Health. 2019 Oct 28;16(21):4154. doi: 10.3390/ijerph16214154 (PMC6862522; doi:10.3390/ijerph16214154)

Supplementary materials

Table S1. Change in birthweight (g) for a 1 μg/m^3 increase in the average $PM_{2.5}$ during 1st, 2nd and 3rd pregnancy trimester, and for prenatal homelessness and the Special Supplemental Nutrition Program for Women, Infants and Children (WIC) participation status in Boston-based Children's HealthWatch cohort (N = 3366).

Variables	Change in birthweight (g) (95% CI)
Average $PM_{2.5}$ during 1st trimester	4.8 (−7.2, 16.9)
Average $PM_{2.5}$ during 2nd trimester	−8.5 (−19.3, 2.3)
Average $PM_{2.5}$ during 3rd trimester	−0.6 (−13.3, 12.2)
Ever homeless or live in shelter during pregnancy with this child	−55.7 (−97.8, −13.7)
Prenatal WIC participation with this child	36.1 (−7.3, 79.4)

Note: adjusted for maternal age, body mass index (BMI), race/ethnicity, nativity, education, smoking history, insurance, marital status, child gestational age and sex, seasonality, and median household income.

Table S2. Change in birthweight (g) for a 1 μg/m³ increase in the average $PM_{2.5}$ during 2nd trimester across maternal and child characteristics in Boston-based Children's HealthWatch cohort (N = 3366).

Groups	Change in birthweight (g) (95% CI)
Maternal nativity	
US born	–5.1 (–18.5, 8.2)
Immigrant	–14.4 (–31.7, 2.9)
Maternal Race/Ethnicity	
White, non-Hispanic	14.0 (–20.1, 48.1)
Hispanic	–8.8 (–27.5, 10.0)
Black, non-Hispanic	–15.2 (-29.4, –0.9)
Other	23.1 (–42.5, 88.6)
Maternal Education	
Some high school or less	–15.4 (-39.0, 8.2)
High school	–11.6 (–30.1, 6.9)
Tech School/College Graduate/Master's	–3.8 (–18.9, 11.2)
Maternal Smoking	
Ever Smoked in the past 5 years	15.9 (–4.3, 36.2)
No smoking	–17.3 (–29.7, –4.8)
Marital status	
Single	–13.8 (–28.0, 0.4)
Married/Partnered/Cohabitating	–4.3 (–22.4, 13.8)
Separated/Divorced	5.1 (–26.2, 36.5)
Child sex	
Male	–12.5 (–27.0, 2.0)
Female	–4.0 (-19.4, 11.3)
Prenatal Homelessness	
No	–8.1 (–19.7, 3.5)
Yes	-10.8 (–37.4, 15.9)
WIC Participation	
No	–18.3 (–45.6, 9.1)
Yes	–6.9 (–18.5, 4.7)

Figure S1. Concentration-response function of the exposure to average $PM_{2.5}$ during 2nd trimester (μg/m^3) on change in birthweight (g) in Boston-based Children's HealthWatch cohort (N = 3366).

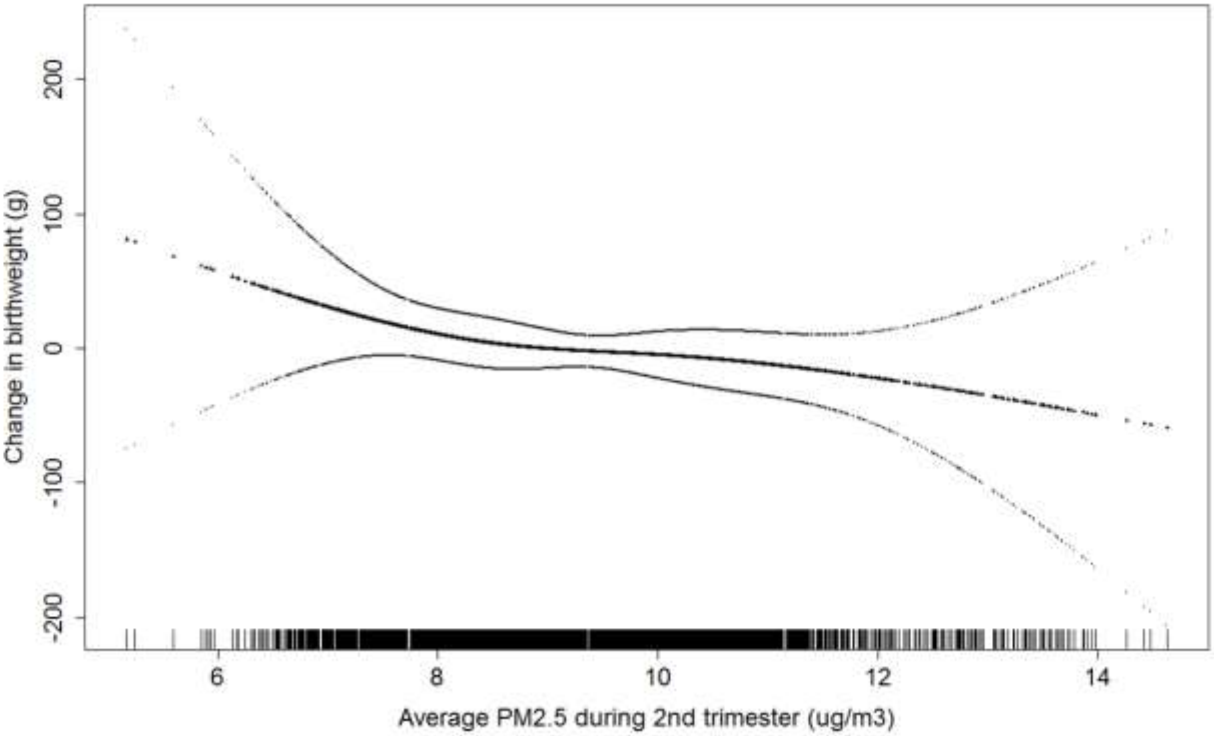

Figure S2. Concentration-response function of the exposure to average $PM_{2.5}$ during 1st trimester ($\mu g/m^3$) on change in birthweight (g) in Boston-based Children's HealthWatch cohort (N = 3366).

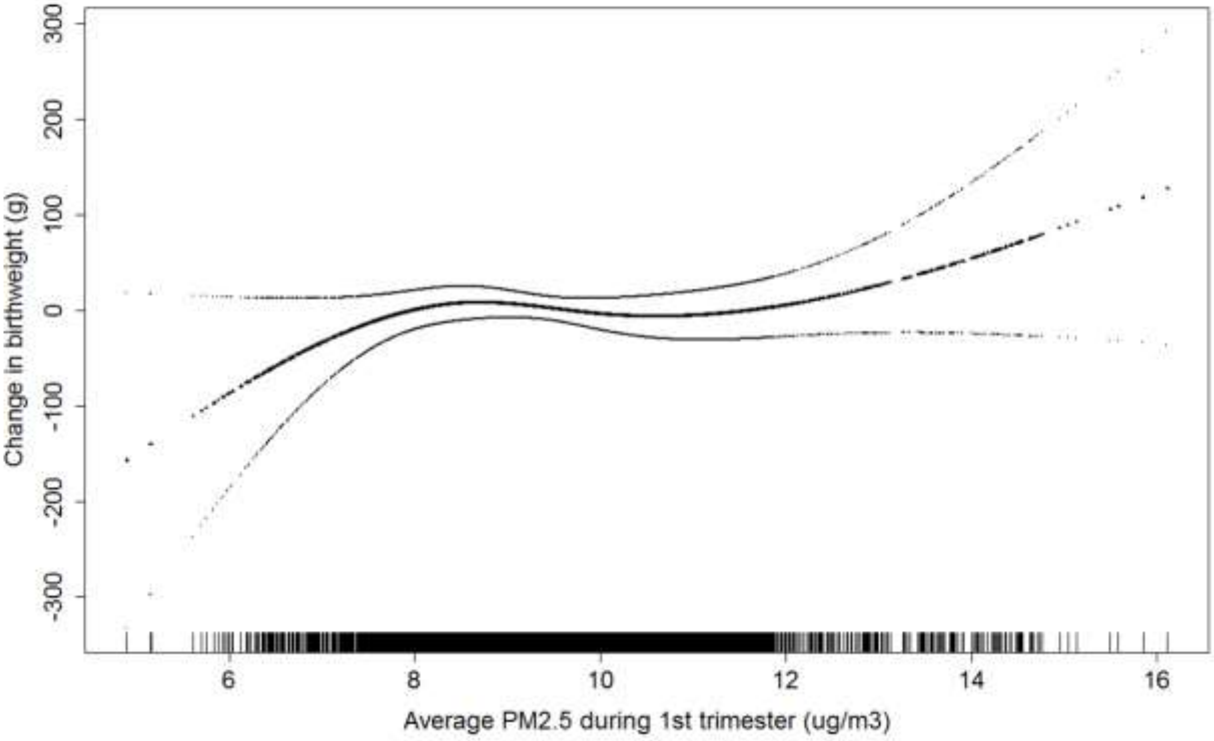

Figure S3. Concentration-response function of the exposure to average $PM_{2.5}$ during 3rd trimester (μg/m3) on change in birthweight (g) in Boston-based Children's HealthWatch cohort (N = 3366)

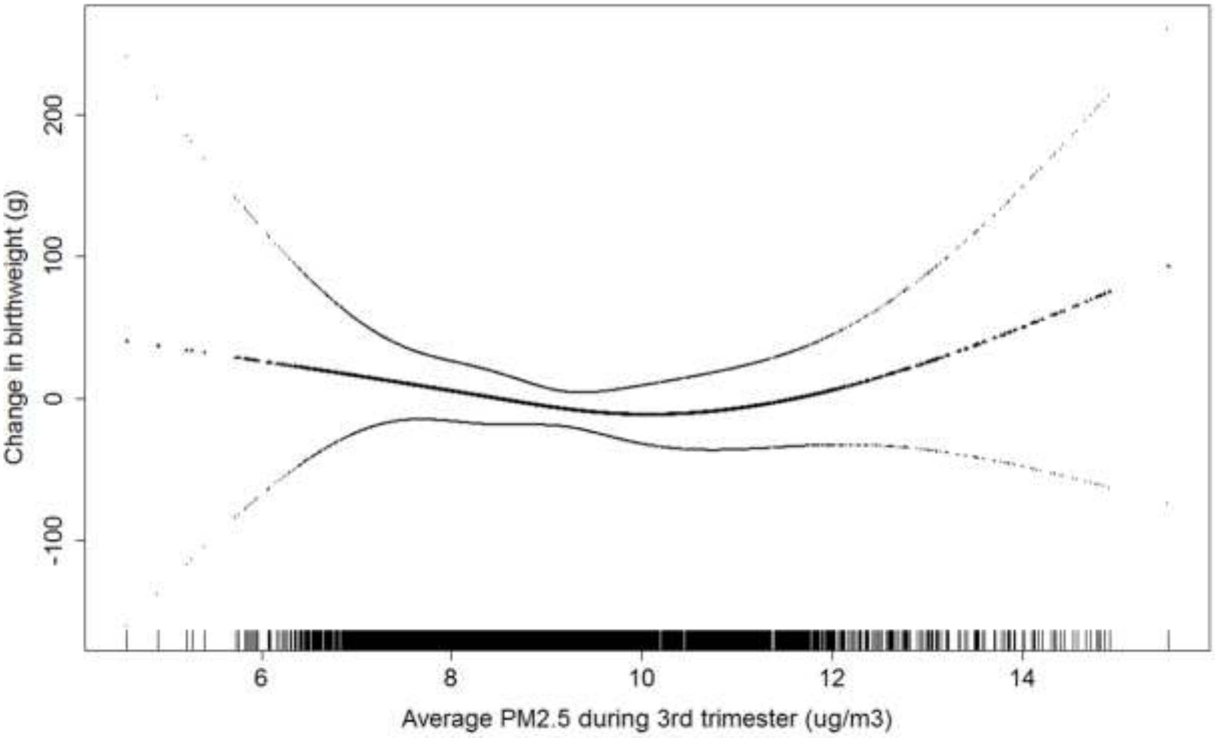

Supplement: Supplementary file 1 [file ijerph-16-04154-s001.pdf]
